# High Throughput Gene Expression Analysis Identifies Reliable Expression Markers of Human Corneal Endothelial Cells

**DOI:** 10.1371/journal.pone.0067546

**Published:** 2013-07-02

**Authors:** Zhenzhi Chng, Gary S. L. Peh, Wishva B. Herath, Terence Y. D. Cheng, Heng-Pei Ang, Kah-Peng Toh, Paul Robson, Jodhbir S. Mehta, Alan Colman

**Affiliations:** 1 A*STAR Institute of Medical Biology, Singapore, Singapore; 2 Tissue Engineering and Stem Cell Group, Singapore Eye Research Institute, Singapore, Singapore; 3 Genome Institute of Singapore, Singapore, Singapore; 4 Singapore National Eye Centre, Singapore, Singapore; 5 Department of Clinical Sciences, Duke-NUS Graduate Medical School, Singapore, Singapore; University of Bristol, United Kingdom

## Abstract

Considerable interest has been generated for the development of suitable corneal endothelial graft alternatives through cell-tissue engineering, which can potentially alleviate the shortage of corneal transplant material. The advent of less invasive suture-less key-hole surgery options such as Descemet’s Stripping Endothelial Keratoplasty (DSEK) and Descemet’s Membrane Endothelial Keratoplasty (DMEK), which involve transplantation of solely the endothelial layer instead of full thickness cornea, provide further impetus for the development of alternative endothelial grafts for clinical applications. A major challenge for this endeavor is the lack of specific markers for this cell type. To identify genes that reliably mark corneal endothelial cells (CECs) *in vivo* and *in vitro*, we performed RNA-sequencing on freshly isolated human CECs (from both young and old donors), CEC cultures, and corneal stroma. Gene expression of these corneal cell types was also compared to that of other human tissue types. Based on high throughput comparative gene expression analysis, we identified a panel of markers that are: i) highly expressed in CECs from both young donors and old donors; ii) expressed in CECs *in vivo* and *in vitro*; and iii) not expressed in corneal stroma keratocytes and the activated corneal stroma fibroblasts. These were SLC4A11, COL8A2 and CYYR1. The use of this panel of genes in combination reliably ascertains the identity of the CEC cell type.

## Introduction

The human corneal endothelium (CE), comprised of a monolayer of hexagonal corneal endothelial cells (CECs) attached to a basement Descemet’s membrane (DM) composed of collagen, forms a selective barrier between the anterior chamber of the eye and the corneal stroma. CECs are the most metabolically active cells in the cornea expressing fluid pumps that actively move fluid from the stroma back into the anterior chamber of the eye. The dynamic balance between the “leaky” barrier and constant pump activity maintains corneal deturgescence, thereby keeping the cornea transparent. Corneal blindness is often due to endothelial dysfunction and is the second leading cause of visual impairment [1].

Human CECs have limited proliferative capacity within the eye [2], [3]. Hence, in order to replace dead or damaged CECs, existing cells spread out to maintain the functional integrity of the CE [4], [5]. In cases of severe cell loss due to genetic corneal endothelial dystrophy or trauma, decompensation of the CE may occur, reducing its capacity to pump fluid out of the stroma, resulting in stromal and epithelial edema, loss of corneal clarity and visual acuity, and eventually bullous keratopathy [1]. The conventional solution to restore vision is through cornea transplantation. However, there is a global shortage of transplant-grade donor cornea. This shortage is expected to worsen as the demand for corneal transplantation increases with an aging global population. Therefore, considerable interest has been generated for the development of suitable endothelial grafts through tissue engineering, which can potentially alleviate the shortage of corneal transplant material.

With the introduction of surgical procedures such as Descemet’s Stripping Endothelial Keratoplasty (DSEK) and Descemet’s Membrane Endothelial Keratoplasty (DMEK), less invasive, key-hole surgery options for the selective replacement of the CE layer are now possible [6], [7], [8]. The advent of such surgical procedures provides further impetus for the development of alternative endothelial grafts – either by means of *ex vivo* expansion of human CECs, or by *de novo* generation of CECs from pluripotent human embryonic stem cells, induced pluripotent stem cells or multipotent adult stem cells, which are unlimited sources of cells. However, a major obstacle to such endeavors is the lack of specific markers for CECs, resulting in an inability to definitively identify such putative stem cell-derived CECs.

Currently, the most commonly used markers in the characterization of cultivated CECs include ZO-1 [9], [10], a tight junction protein involved in signal transduction at cell-cell junctions, and Na^+^/K^+^-ATPase [11], an essential enzyme involved in the active transport of ions across the CE. Although the co-expression of both proteins indicates the presence of key components of CE fluid transport function, it is not a definite indication of the identity of CECs because both ZO-1 and Na^+^/K^+^-ATPase are ubiquitously expressed in many other cell types [12], [13], [14], [15].

This paper presents a thorough gene expression analysis of CECs and proposes a panel of markers that reliably identifies CECs *in vivo* and *in vitro*. We used RNA-sequencing (RNA-seq) [16] and High Throughput Quantitative Polymerase Chain Reaction (HT-PCR), together with various Bioinformatics platforms such as BioGPS (http://biogps.org), DAVID Functional Annotation Clustering Tool [17] and PANTHER Classification System [18], to select a panel of markers based on the following criteria:

1. Highly expressed in CECs from young and old donors:
2. Expressed in CECs *in vivo*, as well as in *ex vivo* CEC cultures;
3. Ideally not expressed in other cell types;
4. Not expressed in corneal stroma keratocytes or activated corneal stroma fibroblasts.

## Results

To identify markers for CECs, global gene expression analysis of CECs stripped from donor cornea along with the Descemet’s membrane (CEC-DM) was carried out using RNA-seq [19]. Gene lists generated were analyzed using DAVID Functional Annotation Clustering Tool [17] and PANTHER Classification System [18] to identify over-represented ontology groups and molecular pathways.

### Identification of Genes most Highly Expressed in CECs

**Figure 1:**
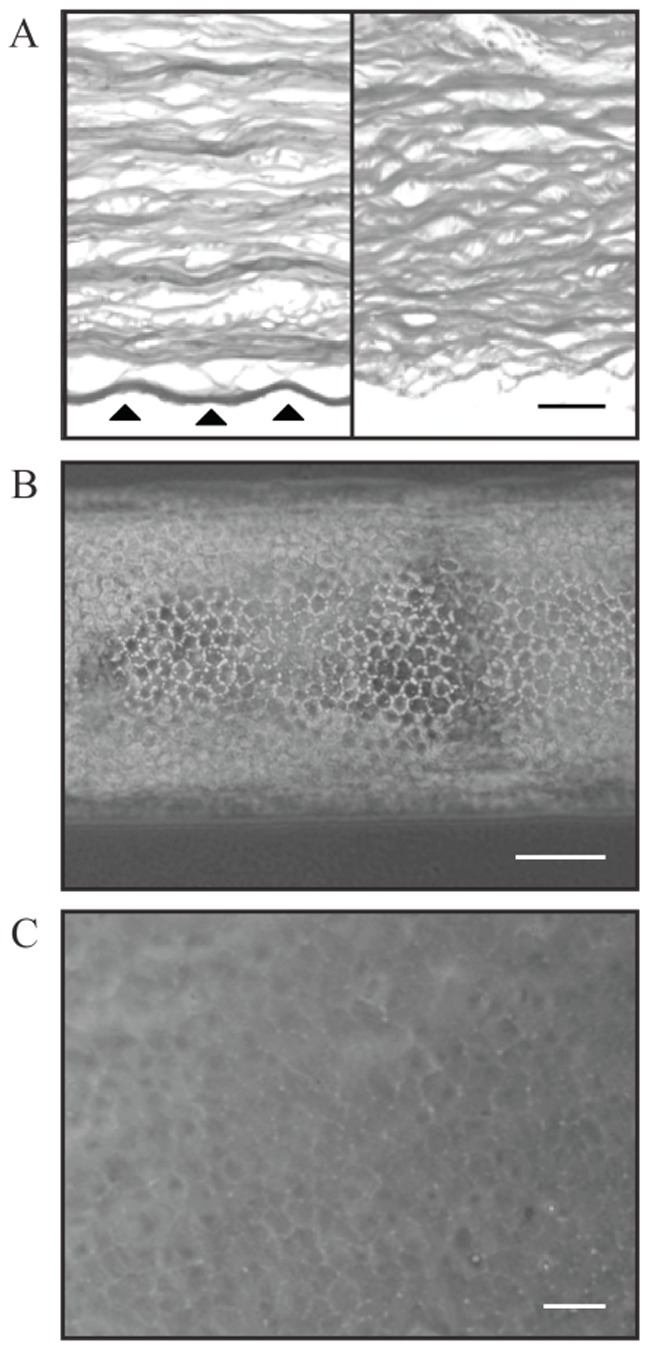
The human corneal tissue, the isolated endothelium and the cultured corneal endothelial cells. A. Corneal stroma with an intact Descemet’s membrane (DM), arrowed (left) and a corneal stromal without the DM layer (right). B. Peeled CEC-DM complex in a typical DM-roll, with the endothelium on the outside. C. Confluent culture of human CECs at the second passage. Scale bars: 100 µm.

Gene expressions of the following samples were analyzed using RNA-sequencing: 1) CEC-DM pooled from 5 young donors; 2) CEC-DM pooled from 5 old donors; 3) CEC cultures and 4) corneal stroma pooled from 5 young donors (Figure 1). Description of the isolation process can be found in Materials and Methods. Full dataset for the RNA-sequencing performed is found in Table S1.

**Figure 2:**
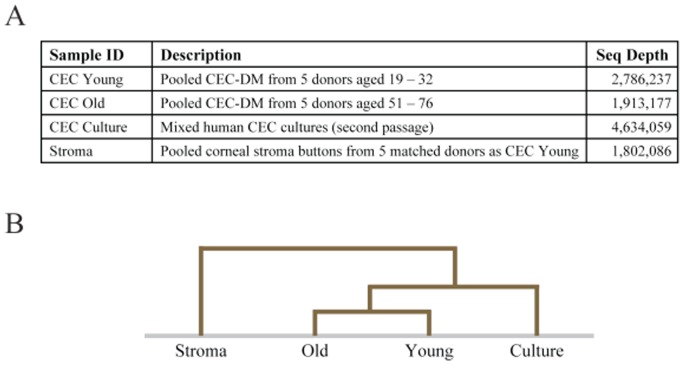
RNA-seq comparing gene expression of young and old CEC-DM, CEC culture and corneal stroma. A. Sequencing depth (total uniquely mapping reads) of each sample. B. Hierarchical clustering shows that the young and old CECs cluster closer to each other, followed by CEC culture, and lastly the corneal stroma.

The sequence depth ranged from 1.8 million to 4.6 million reads (Figure 2A). Hierarchical clustering showed that the young and old CEC-DMs cluster closest to each other, followed by CEC culture, and lastly the corneal stroma (Figure 2B). This indicates that the two CEC-DM samples are closer in gene expression than they are to the CEC culture, and that the corneal stroma sample has the greatest gene expression difference compared to the other 3 samples.

We first sought to identify the genes most highly expressed in the young CECs. The top 20 genes expressed in CECs include those that play a role in cellular metabolism (ENO1, GAPDH, CA3, LDHA, ALDOA, ATP5B, ATP5A1), and genes important for trans-membrane transport (SLC2A1, ATP5B, ATP1A1, ATP5A1) (Table 1). Some of these genes have also been previously identified to be highly expressed in CECs. CA3, PTGDS, LDHA, MGP, and C4orf49 have been identified by Sakai et al. [20], while ENO1, GAPDH, and PTGDS have been noted by Gottsh et al. [21] to be among the top 50 most highly expressed genes in CECs.

**Table 1:** List of top 20 most highly expressed genes in human corneal endothelium.

Gene	Description	PreviouslyIdentified
ENO1	Enzyme important for glycolysis; also functions as a structural lens protein.	Gottsch 2003
GAPDH	Enzyme important for glycolysis.	Gottsch 2003
CA3	Enzyme important for cellular metabolism and carbon dioxide transport; reversible hydration of carbon dioxide.	Sakai 2002
MYOC	Secreted glycoprotein expressed in trabecular meshwork and ciliary body; participates in regulating intraocular pressure.	
SLC2A1	Facilitative glucose transporter.	
PTGDS	Synthesizes prostaglandin PGD2; PGD2 lowers intraocular pressure and triggers inflammatory effects on conjunctiva.	Sakai 2002Gottsch 2003
IER3	Protects cells from Fas- or tumor necrosis factor type alpha-induced apoptosis.	
VIM	Organizer proteins involved in attachment, migration, signaling; mutations in this gene causes a dominant, pulverulent cataract.	
TPT1	Calcium binding and microtubule stabilization.	
ATP5B	Mitochondrial membrane ATP synthase.	
LDHA	Enzyme important for glycolysis.	Sakai 2002
TSPAN6	Mediates signal transduction events; regulates cell development, activation, growth and motility.	
MGP	Associates with the organic matrix of bone and cartilage. Inhibitor of bone formation. Prevents calcification of the cornea.	Sakai 2002
ALDOA	Enzyme important for glycolysis and gluconeogenesis.	
HERPUD1	Component of the endoplasmic reticulum quality control (ERQC) system.	
C4orf49	Corneal Endothelial Specific Protein 1. Expressed in corneal endothelium, corneal epithelium, cultured CECs, brain, testis, and ovary.	Sakai 2002
RPL3	Component of the large subunit of cytoplasmic ribosomes.	
UBC	Ubiquitin C.	
ATP1A1	Na^+^/K^+^-ATPase responsible for establishing Na^+^ and K^+^ gradients essential for osmoregulation	
ATP5A1	Mitochondrial membrane ATP synthase; produces ATP from ADP in the presence of a proton gradient across the membrane	

Information from description was taken from Genecards.org, unless otherwise specified.

Furthermore, analysis using the DAVID Functional Annotation Clustering Tool showed that the most over-represented ontological groups for the top 200 genes in young CECs are cellular metabolism, regulation of cell death, and membrane transport (Table 2). Taken together, these results describe a cell type that is metabolically active, and have a function in ion and water transport, corroborating with previous descriptions of CECs [1], [22].

**Table 2:** GO analysis using DAVID Functional Annotation (Subset: GOTERM_BP_FAT).

Gene ontology term	%	Genes
Generation of precursor metabolites and energy	28.6	ALDOA, LDHA, ATP5B, ALDOC, PFKP, COX4I1, ATP5G3, TPI1, SLC25A3, GNAS, ATP5A1, PGK1, GAPDH, ENO1
Response to organic substance	18.4	HSP90AB1,FOS,HERPUD1,GLUL,ALDOC, MGP,GNAS,ATP5G3,HSPA8
Glycolysis, catabolic processes	16.3	ALDOA, TPI1, LDHA. ALDOC, PFKP, PGK1, GAPDH, ENO1
Translation	16.3	NACA, RPL8, RPL3, UBC, EIF1, RPL11, RPS6, RPS8
Translational elongation	12.2	RPL8, RPL3, UBC, RPL11, RPS6, RPS8
Nitrogen compound biosynthetic process	12.2	ALDOA, GLUL, ATP5B, ATP1A1, ATP5A1, ATP5G3
Cation transport	12.2	SLC4A11, ATP5B, TPT1, ATP1A1, ATP5A1, ATP5G3
Homeostatic process	12.2	ALDOA, HERPUD1, SLC4A11, ATP5B, TPT1, RPS6
ATP/nucleotide biosynthetic process	10.2	ALDOA, ATP5B, ATP1A1, ATP5A1, ATP5G3
Monovalent inorganic cation transport	10.2	SLC4A11, ATP5B, ATP1A1, ATP5A1, ATP5G3
Proton Transport	8.2	SLC4A11, ATP5B, ATP5A1, ATP5G3
Response to protein stimulus	8.2	HSP90AB1, FOS, HERPUD1, HSPA8
Organic aid biosynthetic process	8.2	TPI1, GLUL, PTGDS, SCD
Response to extracellular stimulus	8.2	FOS, LDHA, COZ4I1, MGP
Cation homeostasis	8.2	HERPUD1, SLC4A11, ATP5B, TPT1
Fructose metabolic process	6.1	ALDOA, ALDOC, PFKP
ATP synthesis coupled proton transport, ion transmembrane transport	6.1	ATP5B, ATP5A1, ATP5G3
Response to unfolded protein	6.1	HSP90AB1, HERPUD1, HSPA8
Fatty acid biosynthetic process	6.1	TPI1, PTGDS, SCD
Oxidative phosphorylation	6.1	ATP5B, ATP5A1, ATP5G3
Aging	6.1	FOS, ALDOC, ATP5G3
One-carbon metabolic process	6.1	FOS, CA12, CA3
Biosynthetic/metabolic processes	4.1	ATP5B, ATP5A1
Protein homotetramerization	4.1	ALDOC, PFKP

Top 200 genes with highest RPM value in young CEC-DM was analyzed in DAVID. GO terms that are over represented are ranked in descending order of percentage of genes representing this category. Related GO terms represented by the same set of genes were combined.

### Analysis of Genes that have been Previously used as CEC Markers

We next examined the expression levels of commonly used CEC markers in our samples. Table 3 shows that most of the markers previously used by other groups are either not expressed in culture (reads per million (RPM) <10), do not differentiate between CECs and stroma (fold change of young/stroma <5), or lack specificity as catalogued in BioGPS. Of the genes listed, COL8A2 and SLC4A11 emerged from our study as the best candidates as markers for CECs, but these too are not ideal. Expression of COL8A2 is high in CECs and low in stroma, but is also present in the retina. SLC4A11 is highly expressed in CECs, not ubiquitously expressed in many cell types (data not shown), but is 6 times lower in expression in CEC cultures than in CEC-DM (Table 3). Therefore, we sought to probe for other candidates that could be used to identify CECs.

**Table 3:** Analysis of CEC markers used in other publications.

	RPM values				Fold change			Flaw			
Gene Symbol	CEC Young	CEC Old	CEC Culture	Corneal Stroma	Young/Old	Young/Culture	Young/Stroma	1	2	3	4
AQP1	233	172	4	506	1.35	54.0	0.5		Y	Y	Y
ATP1A1	2,616	1,627	858	368	1.61	3.1	7.1				Y
CLCN2	13	0	1	4	n/a	11.6	2.8	Y	Y	Y	
CLCN3	15	5	79	5	2.82	0.2	3.0	Y		Y	Y
COL8A2	1,821	1,743	859	107	1.04	2.1	17.0				R
ENO2	495	247	57	104	2.0	8.7	4.8			Y	Y
JAM1	0.80	0.50	3.70	86	1.60	0.22	0.01	Y	Y	Y	Y
NCAD	129	67	94	4	1.92	1.4	33.3				Y
PTGDS	5,547	6,415	183	2,522	0.86	30.2	2.2		?	Y	Y
SLC4A11	2,313	2,940	366	90	0.79	6.3	25.7		?		
SLC4A4	113	221	41	29	0.51	2.8	3.9			Y	Y
VDAC2	248	135	134	172	1.83	1.9	1.4			Y	Y
VDAC3	582	393	299	250	1.48	2.0	2.3			Y	
ZO-1	49	42	8	72	1.17	6.3	0.7	Y	Y	Y	Y

1: Not high in young CECs (RPM<100);

2: Not expressed in culture (RPM<10);

3: Does not differentiate between CEC and stroma (Young/stroma < 5);

4: Lack of specificity as assessed in BioGPS;

Y: Yes;

?: Not conclusive;

R: retina.

### Identification of Genes Important for CEC Function and Physiology

To identify genes important for CEC physiology, genes that are highly expressed in CEC-DM and CEC cultures were selected and analyzed. The list was filtered for genes with RPM>10 in young CEC-DM, old CEC-DM and CEC culture, at least two times higher in young CECs than in corneal stroma, and less than 2-fold difference in expression between young and old CEC. The top 50 genes ranked in descending order of expression in young CEC-DM are shown in Table 4. Each gene was analyzed in BioGPS to obtain information on the expression of each gene across different tissues (data not shown). Genes that are not ubiquitously expressed were further analyzed using high-throughput QPCR to compare the expression of these selected genes in various human tissue types.

**Table 4:** List of top 50 highly expressed genes found in human corneal endothelium that are 2-fold higher in CECs than in stroma, and less than 2-fold difference between young and old corneal endothelium.

	RPM Values				Fold Changes	
Gene symbol	Young	Old	Culture	Stroma	Young/Stroma	Young/Old
ENO1	35,947	25,205	19,318	14,237	2.5	1.4
GAPDH	8,083	6,6498	6,598	2,401	3.4	1.2
CA3	7,804	6,267	166	207	37.7	1.2
MYOC	6,780	10,193	8,272	915	7.4	0.7
SLC2A1	5,598	4,593	3,491	1,921	2.9	1.2
PTGDS	5,547	6,416	184	2,522	2.2	0.9
IER3	5,311	5,829	1,353	1,779	3.0	0.9
ATP5B	4,762	4,413	3,095	1,330	3.6	1.1
TSPAN6	3,860	3,668	1,617	137	28.2	1.1
MGP	3,598	3,419	3,813	345	10.4	1.1
ALDOA	3,421	2,425	2,812	1,557	2.2	1.4
HERPUD1	3,138	2,367	649	862	3.6	1.3
ATP1A1	2,616	1,627	858	368	7.1	1.6
ATP5A1	2,586	1,737	1,640	979	2.6	1.5
GNAS	2,574	2,550	2,112	1,165	2.2	1.0
PGK1	2,469	2,878	2,239	906	2.7	0.9
CA12	2,322	1,756	1,721	123	18.8	1.3
SLC4A11	2,312	2,940	366	90	25.7	0.8
COX4I1	2,265	2,232	1,011	815	2.8	1.0
TSC22D1	2,070	2,038	2,978	865	2.4	1.0
SCD	1,928	1,108	252	54	35.8	1.7
COL8A2	1,821	1,743	859	107	17.0	1.0
PFKP	1,577	1,220	433	158	10.0	1.3
ADM	1,287	1,712	314	491	2.6	0.8
NDUFB8	1,167	997	664	553	2.1	1.2
SFRP1	1,134	1,592	38	125	9.0	0.7
EIF3K	1,070	1,292	648	385	2.8	0.8
RPL15	1,063	1,033	674	451	2.4	1.0
GHITM	1,042	1,081	747	224	4.6	1.0
C5orf62	970	971	472	57	17.1	1.0
AKR1C1	953	729	678	180	5.3	1.3
AKR1B1	892	777	988	229	3.9	1.1
LAMB1	890	825	753	73	12.2	1.1
ID3	834	1,744	1,337	133	6.3	0.5
TUBA4A	831	934	191	390	2.1	0.9
TUFM	767	561	496	302	2.5	1.4
CD83	750	678	72	81	9.3	1.1
SULF2	749	470	791	29	25.5	1.6
NPC2	744	488	1,963	366	2.0	1.5
TMEM66	632	517	844	321	2.0	1.2
MIF	628	773	450	314	2.0	0.8
RGS5	608	930	139	22	27.4	0.7
C6orf48	603	589	652	290	2.1	1.0
NDUFB9	589	869	405	234	2.5	0.7
NDUFB10	583	323	235	98	6.0	1.8
VDAC3	582	393	299	250	2.3	1.5
P4HA2	572	231	603	104	5.5	2.0
ITGB5	571	613	1,333	137	4.2	0.9
NDUFV1	570	554	104	77	7.4	1.0
GRHPR	569	667	768	123	4.6	0.9
ANKH	569	444	687	123	4.6	1.3

Genes are ranked in descending order of RPM values of young CEC-DM. RPM values are rounded off to the nearest integer, and fold change values are rounded off to the nearest decimal place.

**Figure 3:**
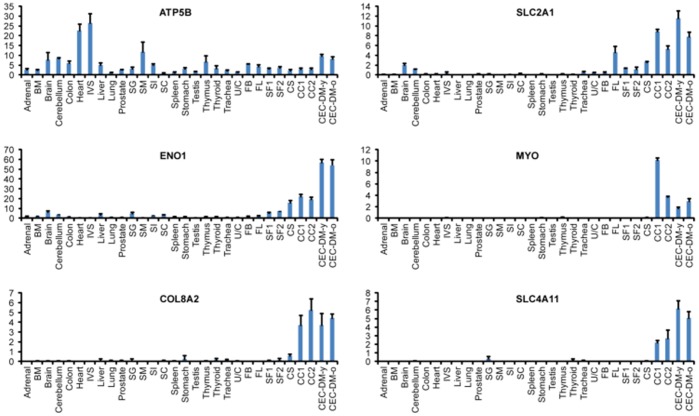
High-throughput QPCR analysis of genes expressed in both CEC-DM and CEC cultures. BM: bone marrow; IVS: interventricular septum; SG: salivary gland; SM: skeletal muscle; SI: small intestine; SC: spinal cord; U/C: uterus/cervix; FB: fetal brain; FL: fetal liver; SF: stromal fibroblast; CC: CEC culture; CEC-DM-y: CEC-DM young; CEC-DM-o: CEC-DM-old.

QPCR results show that ATP5B is expressed in all the tissue types tested. SLC2A1, ENO1, and COL8A2 are highly expressed in CEC cultures and CEC-DM but are also expressed in many other tissue types, albeit at lower levels. MYOC and SLC4A11 on the other hand are highly expressed in CEC cultures and CEC-DM but not in other tissue types (Figure 3).

**Figure 4:**
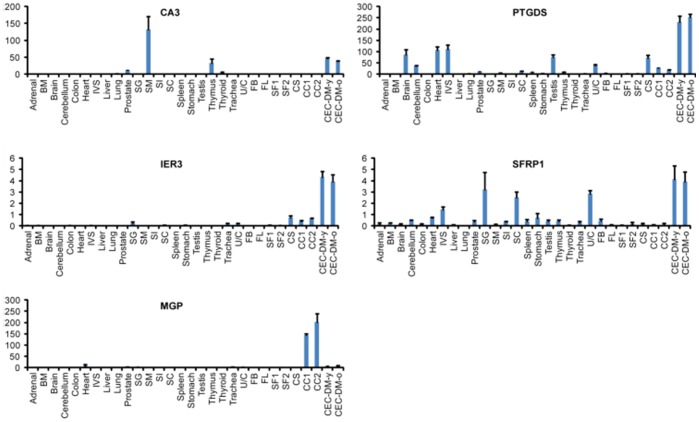
High-throughput QPCR analysis of genes differentially expressed in CEC-DM and CEC cultures. BM:bone marrow; IVS:interventricular septum; SG: salivary gland; SM: skeletal muscle; SI: small intestine; SC: spinal cord; U/C:uterus/cervix; FB:fetal brain; FL:fetal liver; SF:stromal fibroblast; CC:CEC culture; CEC-DM-y:CEC-DM young; CEC-DM-o:CEC-DM-old.

Interestingly, the expression of CA3, PTGDS, IER3 and SFRP1 were high in both young and old CEC-DM, but were lost in CEC cultures, indicating that the expression of these genes are not necessary for CEC survival *in vitro*. While they could be useful for identifying CECs *in vivo*, they should not be used as CEC markers in experiments involving CEC cultures. On the other hand, MGP is expressed much higher in CEC cultures than in CEC-DM and other tissue types (Figure 4).

Taken together, the results above suggest that SLC4A11 and COL8A2 are suitable markers for CECs based on their high expression in CEC-DM and CEC-cultures compared to other cell types. MYOC is not an ideal choice because it is expressed in the trabecular meshwork where it participates in the obstruction of fluid outflow.

### Identifying Genes that are Expressed in CECs but not in Corneal Stroma

There was a trace amount of SLC4A11 and COL8A2 in corneal stroma keratocytes and/or its activated stromal fibroblasts (Figure 3). Therefore, we sought to identify other genes that were not expressed or lowly expressed in corneal stroma. We selected genes that have RPM value of less than 1 in corneal stroma but more than 10 in the other 3 samples (Table 5). Each gene was analyzed in BioGPS (data not shown). Genes that are not ubiquitously expressed were chosen for further analyses.

**Table 5:** Genes not expressed in the corneal stroma.

	RPM Values			
Gene Symbol	CEC Young	CEC Old	CEC Culture	Corneal Stroma
OLFML1	96.55	69.52	19.64	0.55
CYYR1	94.39	15.68	10.14	0.00
HEMK1	91.52	71.61	41.86	0.55
ACAD8	90.09	26.66	25.90	0.55
ATRN	75.01	58.54	48.34	0.55
GNAZ	70.70	70.56	27.84	0.55
HIPK1	70.70	12.02	17.05	0.00
FBP1	60.66	65.34	31.07	0.55
LARS	59.58	26.66	18.99	0.55
GSTZ1	53.84	47.56	71.64	0.00
NUP54	51.32	10.98	10.57	0.55
UBA5	50.96	35.54	13.38	0.55
UBR7	50.25	35.02	58.70	0.55
KIAA1549	47.02	36.59	12.08	0.55
SRGAP2	46.30	17.25	29.13	0.00
NUAK1	45.22	15.16	26.76	0.55
ZFPL1	43.79	49.13	90.85	0.55
UPRT	43.07	28.23	15.11	0.55
KIF3C	42.71	12.54	11.01	0.55
UPRT	42.35	28.23	15.11	0.55

Gene list is filtered for RPM>10 for CEC Young, CEC Old and CEC Culture, and RPM<1 for corneal stroma. Genes are ranked in descending order of RPM values of CEC Young. All values are rounded off to 2 decimal places.

**Figure 5:**
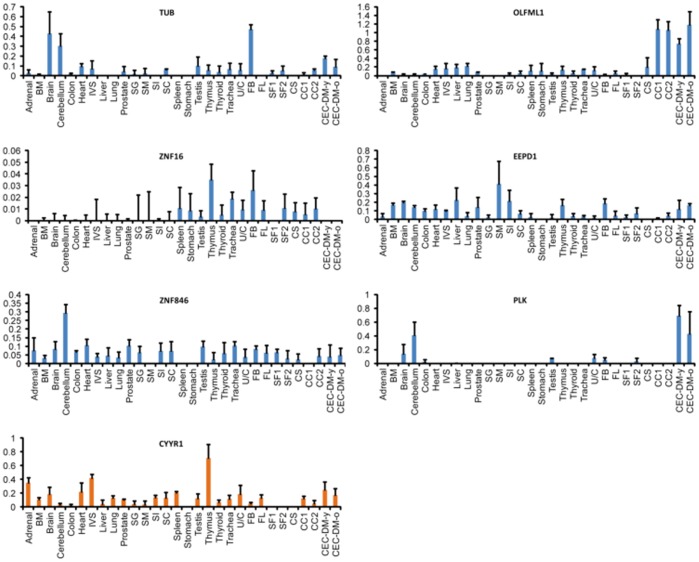
High-throughput QPCR analysis of genes lowly or not expressed in corneal stroma. BM: bone marrow; IVS: interventricular septum; SG: salivary gland; SM: skeletal muscle; SI: small intestine; SC: spinal cord; U/C: uterus/cervix; FB: fetal brain; FL: fetal liver; SF: stromal fibroblast; CC: CEC culture; CEC-DM-y: CEC-DM young; CEC-DM-o: CEC-DM-old.

High throughput QPCR was first performed using Fluidigm Biomark system to compare expression of these genes across different human tissue types. Results show that of all the genes analyzed, CYYR1 was the only gene that was expressed in CEC-DM and CEC cultures, but not expressed in corneal stroma and stroma fibroblast cultures (Figure 5).

### Confirming the Stability of Expression of SLC4A11, COL8A2 and CYYR1 across Multiple Biological Samples

**Figure 6:**
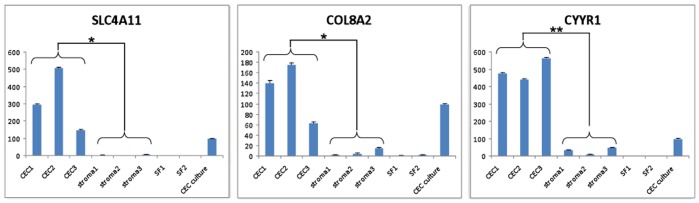
QPCR validation of gene expression in CEC-DM andcorneal stroma (keratocytes) from 3 individual donors, corneal stromal fibroblast cultures from 2 individual donors and CEC culture. CEC: CEC-DM; stroma: corneal stroma (keratocytes); SF: stromal fibroblast. Student’s t-tests (two-tailed assuming non-equal variance): One asterisk indicates p<0.05; two asterisks indicate p<0.01.

QPCR analysis was performed using a 7500 Fast Real Time PCR machine to compare the expression of SLC4A11, COL8A2, and CYYR1 in CECs and corneal stroma from 3 individual donors. Expression of CYYR1 was similar in all three donors (average ± standard deviation: 495±54), while that of SLC4A11 and COL8A2 varied slightly (average ± standard deviation: 318±181 and 126±57 respectively). On average, expression of SLC4A11 was 84-fold higher in CECs than in stroma (*p*<0.05), while expression of COL8A2 and CYYR1 was 16-fold higher in CECs than in corneal stroma (*p*<0.05 and *p*<0.01 respectively). Expression of all three genes was negligible in stromal fibroblast cultures obtained from 2 individual donors (Figure 6). Taken together, the combination of SLC4A11, COL8A2 and CYYR1 present a useful panel of genes to differentiate between CECs and corneal stroma/stroma fibroblast.

### Gaining Insight into CEC Physiology from Gene Expression

The transparency of the cornea is predominantly attributed to the ion and fluid transport capacity of CECs. We were therefore interested to examine the expression of transporters in CECs (Table S2). Genes that are classified as transporters under the PANTHER classification system were extracted. The gene list was filtered for RPM>10 in young CEC-DM, old CEC-DM and CEC culture, at least two times higher in young CECs than in corneal stroma, and less than 2-fold difference in expression between young and old CEC. Table 6 shows the list of transporters that fit these criteria, ranked in descending order of expression in young CECs.

**Table 6:** Expression of transporter-associated genes found in corneal endothelium, cultured corneal endothelial cells and corneal stroma.

	RPM Values				Fold Changes	
Gene symbol	Young	Old	Culture	Stroma	Young/Stroma	Young/Old
SLC2A1	5598	4593	3491	1921	2.9	1.2
ATP5B	4762	4413	3095	1330	3.6	1.1
ATP1A1	2616	1627	858	368	7.1	1.6
ATP5A1	2586	1737	1640	979	2.6	1.5
SLC4A11	2313	2940	366	90	25.7	0.8
SLC25A4	540	536	396	116	4.7	1.0
ATP6V1E1	402	403	479	188	2.1	1.0
PCOLCE	397	601	317	42	9.4	0.7
ATP5G1	236	279	189	113	2.1	0.8
SLC22A4	231	143	35	5	46.3	1.6
SORT1	216	237	214	8	27.8	0.9
SLC15A4	195	358	104	43	4.6	0.5
SVEP1	182	257	390	11	16.4	0.7
ATP5J	162	141	129	23	6.9	1.2
SLC25A11	132	85	152	23	5.8	1.6
TAP1	128	192	126	6	20.9	0.7
ATP6V0D1	126	113	245	62	2.0	1.1
SLC4A4	113	221	41	29	3.9	0.5
ATP6V1D	109	161	374	43	2.5	0.7
SLC35E1	107	71	63	11	9.6	1.5
PAPPA	79	97	89	4	20.2	0.8
SLC2A3	73	77	18	32	2.3	0.9
LRPPRC	64	54	37	5	12.9	1.2
SLC 25A13	59	103	16	12	5.1	0.6
SLC39A6	59	83	27	14	4.1	0.7
TM9SF2	55	30	28	19	2.8	1.8
COX11	55	94	44	24	2.2	0.6
VPS33B	52	63	33	4	13.4	0.8
TM9SF1	51	29	191	4	13.1	1.7
SEC61A2	45	87	15	2	20.1	0.5
SLC5A3	43	40	22	13	3.3	1.1
SLC16A2	38	58	304	11	3.6	0.6
PITPNM1	36	46	33	1	32.7	0.8
ABCF3	36	36	60	14	2.6	1.0
TOM1L2	34	24	26	4	7.7	1.4
SLC26A11	33	28	12	1	30.1	1.2
SLC35B3	29	36	113	2	17.2	0.8
SLC16A3	28	38	86	4	6.3	0.7
SLC4A2	27	51	85	6	4.8	0.5
CACNB3	27	40	41	1	47.9	0.7
AP3D1	24	32	114	3	7.2	0.7
IPO9	22	11	77	6	3.5	1.9
SLC45A1	18	16	13	1	16.5	1.1

Transporters expressed in CECs that are at least 2 times higher in young CEC-DM than in stroma, and less than 2 fold difference in expression between young and old CEC-DM. Genes are ranked in descending order of RPM values of young CEC-DM. RPM values are rounded off to the nearest integer, and fold change values are rounded off to the nearest decimal place.

The high throughput gene expression analyses performed can also be used to further understand the signaling pathways that govern CEC physiology. To achieve this aim, we looked at the expression of cytokine receptors (Table S3) and transcription factors (Table S4) in CECs. Genes that are classified as cytokine receptors or transcription factors under the PANTHER classification system were extracted. The gene lists were filtered for RPM>10 in young CEC-DM, old CEC-DM and CEC culture, at least two times higher in young CECs than in corneal stroma, and less than 2-fold difference in expression between young and old CEC. Only 3 cytokine receptors pass the selection criteria (Table 7). Table 8 shows a similar list of transcription factors expressed in CECs.

**Table 7:** Gene expression of cytokine receptors found in corneal endothelium, cultured corneal endothelial cells and corneal stroma.

	RPM Values				Fold Changes	
Gene symbol	Young	Old	Culture	Stroma	Young/Stroma	Young/Old
TGFBR3	135	130	11	44	3.1	1.0
IL13RA1	78	95	50	16	4.8	0.8
IL6ST	43	76	25	9	4.5	0.6

Cytokine receptors expressed in CECs that are at least 2 times higher in young CEC-DM than in stroma, and less than 2 fold difference in expression between young and old CECs. Genes are ranked in descending order of RPM values of young CEC-DM. RPM values are rounded off to the nearest integer, and fold change values are rounded off to the nearest decimal place.

**Table 8:** Gene expression of transcription factors found in corneal endothelium, cultured corneal endothelial cells and corneal stroma.

	RPM Values				Fold Changes	
Gene symbol	Young	Old	Culture	Stroma	Young/Stroma	Young/Old
KLF10	566	719	80	195	2.9	0.8
SNURF	539	272	547	235	2.3	2.0
PER1	300	192	57	93	3.2	1.6
ILF2	290	435	379	62	4.7	0.7
MAFF	289	297	36	119	2.4	1.0
PITX2	288	567	377	11	27.3	0.5
TFAP2B	277	244	110	5	45.5	0.9
SF1	184	244	115	32	5.8	0.5
SMAD3	180	275	55	38	4.7	0.7
ERG	127	102	64	24	5.3	1.2
STAT1	125	212	166	57	2.2	0.6
SMYD3	122	109	78	2	55	1.1
TCEB3	118	93	99	6	21.3	1.3
EPAS1	118	156	90	49	2.4	0.8
CAND1	118	183	85	49	2.4	0.6
CAND1	118	183	85	49	2.4	0.6
REPIN1	113	64	103	12	9.7	1.8
NFKBIZ	107	110	17	52	2.0	1.0
KBTBD4	103	64	71	12	8.4	1.6
L3MBTL2	102	71	23	37	2.7	1.4
TCEB2	100	128	95	42	2.4	0.8
POLR3H	95	53	132	35	2.7	1.8
KLF9	89	141	47	19	4.7	0.6
GTF2IRD1	81	51	23	1	72.8	1.6
COPS5	77	88	365	33	2.3	0.9
RXRB	75	93	50	8	9.1	0.8
ZNF3	71	84	26	10	7.1	0.8
ZNF143	67	72	49	31	2.2	0.9
YAP1	66	67	35	6	10.8	1.0
ING1	65	96	25	2	29.4	0.7
ZBTB47	65	36	25	1	58.5	1.8
ZKSCAN5	63	34	22	2	28.5	1.8
FOXC1	60	113	24	9	6.7	0.5
ZNF664	59	50	53	28	2.1	1.2
ZMYM4	58	61	19	2	34.9	1.0
SPRYD4	57	36	47	11	5.1	1.6
TCEAL8	57	54	58	13	4.4	1.0
ZFHX4	56	81	44	2	33.4	0.7
E2F4	55	35	35	27	2.1	1.6
RUVBL2	55	95	153	5	10.9	0.6
LMX1B	54	38	57	2	24.4	1.4
TARDBP	52	34	44	8	6.3	1.5
TBPL1	51	54	18	2	30.6	0.9
GZF1	48	75	60	3	14.3	0.6
ZFPL1	44	49	91	1	78.9	0.9
HCFC2	37	61	16	2	22	0.6
ZMAT3	36	22	33	8	4.6	1.6
PCGF3	35	41	13	4	9.0	0.8
RBX1	34	18	49	14	2.4	1.9
STAT2	33	57	102	8	4.2	0.6
CTBP2	32	39	38	3	11.6	0.8
ZNF862	31	22	11	2	13.7	1.4
QRICH1	30	30	11	1	54.3	1.0
CREM	27	52	56	3	9.7	0.5
EGFL6	23	32	29	1	21	0.7
RNF14	23	25	19	4	6.0	0.9
RBL2	23	38	19	4	6.0	0.6
NFYC	22	33	13	5	4.4	0.7
CREB3L4	20	19	47	1	36.9	1.1
CTBP1	20	10	17	3	6.0	1.9
MAF	20	21	12	6	3.2	0.9
NFYA	19	14	13	1	34.3	1.4
ZNF740	16	25	24	2	7.1	0.6

Transcription factors expressed in CECs that are at least 2 times higher in young CEC-DM than in stroma, and less than 2 fold difference in expression between young and old CECs. Genes are ranked in descending order of RPM values of young CEC-DM. RPM values are rounded off to the nearest integer, and fold change values are rounded off to the nearest decimal place.

We next performed pathway analysis of the transcription factors using DAVID Functional Annotation. The signaling pathways represented by the expressed transcription factors include TGFβ and Wnt signaling pathways (Table 9). The expression of components of these signaling pathways were further examined (Tables S5 and S6). Interestingly, we found that CECs do not express all SMADs (intracellular mediators of TGFβ signaling). The most highly expressed SMADs (SMAD2, SMAD3 and SMAD4) in CECs are those involved in the Activin/Nodal branch of TGFβ signaling (Table 10). Such information provides further clues for future work to delineate the signaling pathways controlling CEC physiology and gene expression.

**Table 9:** Pathway analysis of transcription factors via DAVID Functional Annotation.

Term	%	Genes
Pathways in cancer	12.1	CTBP1, CTBP2, EPAS1, RXRB, TCEB2, SMAD3, STAT1, RBX1
TGF-beta signaling pathway	7.6	E2F4, RBL2,SMAD3,RBX1,PITX2
Cell cycle	6.1	E2F4, RBL2,SMAD3,RBX1
Wnt signaling pathway	6.1	CTBP1, CTBP2, SMAD3, RBX1
Renal cell carcinoma	4.6	EPAS1, TCEB2, RBX1
Chronic myeloid leukemia	4.6	CTBP1, CTBP2, SMAD3

Transcription factors expressed in young CEC-DM, old CEC-DM and CEC culture (RPM>10) that are more than two-fold higher in young CEC-DM versus stroma, were analyzed in DAVID. Pathways that are over represented are ranked in descending order of percentage of genes representing this category.

**Table 10:** Expression of SMADs in corneal endothelium, cultured corneal endothelial cells and corneal stroma.

	RPM Values			
GeneSymbol	Young	Old	Culture	Stroma
SMAD3	180	275	55	38
SMAD2	106	45	82	131
SMAD4	28	11	35	0
SMAD5	23	0	3	2
SMAD9	14	0	0	0
SMAD7	5	46	1	3
SMAD6	1	0	0	0
SMAD1	0	5	0	0

Genes are ranked in descending order of RPM values of young CEC-DM. RPM values are rounded off to the nearest integer.

## Discussion

### Expression Markers for CECs

The challenges and important Clinical Quality Assurance (CQA) points that should be taken into consideration for CEC therapy include identity, potency, purity, and safety. To date, no one specific gene has been identified as a marker of CECs. While generic markers such as ZO-1 and Na^+^/K^+^-ATPase indicate the presence of functional fluid transport, they do not definitively identify CECs. Indeed, co-expression of ZO-1 and Na^+^/K^+^-ATPase has been observed in mammalian blastocyst [23], [24], and in various adult tissues including intestinal epithelium [25] and retinal pigmented epithelium [26]. The use of our proposed panel of genes (SLC4A11, COL8A2 and CYYR1) together ascertain the identity of the CEC cell type, thereby fulfilling the first CQA point that renders the cell suitable for clinical use.

Both SLC4A11 and COL8A2 are known to play important functions in the cornea. SLC4A11 is a multi-pass membrane protein important for sodium-mediated fluid transport. It prevents morphological abnormalities of the cornea caused by increased sodium chloride concentrations in the stroma. Mutations in SLC4A11 have been known to cause Harboyan Syndrome (congenital corneal endothelial dystrophy with progressive perceptive deafness) [27], Corneal Endothelial Dystrophy Type 2 (bilateral corneal dystrophy characterized by corneal opacification and nystagmus) [28], and Fuchs Endothelial Dystrophy (ocular disorder characterized by focal wart-like guttata that arise from DM and develop in the central cornea, epithelial blisters, reduced vision and pain) [29].

COL8A2 encodes the alpha 2 chain of type VIII collagen. Type VIII collagen is a major component of the DM [30]. Mutations in COL8A2 have been shown to cause Fuchs Endothelial Corneal Dystrophy and Posterior Polymorphous Corneal Dystrophy Type 2 [31], [32], [33], [34], [35], [36], [37].

Little is known about CYYR1, also called cysteine and tyrosine-rich protein 1. It is a recently identified gene located on human chromosome 21 whose product has no similarity to any known protein and is of unknown function [38]. Analysis of expressed sequence tags revealed high human CYYR1 expression in cells belonging to the diffuse neuroendocrine system, which may be the origin of neuroendocrine tumors [39].

### Differentiating between Corneal Endothelial Cells and Corneal Stroma Cells

The inclusion of CYYR1 as part of our panel to identify CECs is crucial, because it helps differentiate CECs from corneal stroma keratocytes and the serum-activated keratocytes known as corneal stroma fibroblasts. This is especially important for researchers studying CEC explants and CEC cultures as slow-growing CECs can be contaminated with, and over-taken by the highly proliferative corneal stroma fibroblasts that may be co-isolated during the isolation of CEC for culture [40]. In addition, CECs are the closest to corneal stroma keratocytes in terms of embryonic origin. During development, both cell types are derived from cranial neural crest cells, which migrate and settle in the head mesenchyme. In chick and human, the first wave of migration of mesenchymal cells forms the endothelial layer on the inner surface of the cornea, adjacent to the lens. Shortly thereafter, a second wave of mesenchymal cells invades the primary stroma where the mesenchymal cells differentiate into keratocytes [41], [42]. Therefore, a reliable panel of genes to identify *de novo* CECs generated from pluripotent stem cells must have considerably lower expression in stromal keratocytes/fibroblasts compared to CECs.

### Signaling Pathways Controlling CECs Physiology

The high throughput gene expression analysis performed is not only useful for identifying markers for CECs, but also provides insight into the signaling pathways controlling CEC physiology. Examination of the cytokine receptors and transcription factors expressed revealed that TGFβ (in particular the Activin/Nodal branch) and Wnt signaling pathways are active in CECs.

Indeed, exogenous TGFβ in the aqueous humor has been shown to suppress S-phase entry in CEC cultures, suggesting that TGFβ could play a role in the inhibition of proliferation of CECs [43]. In the developing eye, TGFβ from the lens has been shown to control the development of the neural crest–derived eye structures (corneal stroma, corneal endothelium, anterior iris and trabecular meshwork) [44]. The migration of periocular mesenchyme into the region of the prospective corneal stroma and endothelium is attributed to a response to TGFβ signals from the lens. Furthermore, TGFβ2 released from the lens is required for the expression of transcription factors PITX2 and FOXC1 [44], whose expressions in periocular mesenchyme are necessary for the proper formation of the anterior segment of the eye [45].

Wnt signalling has also been implicated in the development of CECs. Canonical Wnt signalling is required for maintenance of PITX2 expression in ocular neural crest cells during cornea development [46]. Interestingly, the Wnt antagonist DKK2 is an essential downstream target of the PITX2 homeodomain transcription factor in neural crest during eye development, to provide a mechanism to locally suppress canonical Wnt signaling activity during eye development [47].

Therefore, manipulation of TGFβ and Wnt signaling pathways in CECs could help improve conditions for *ex vivo* culture of CECs, and direct differentiation of pluripotent stem cells or neural crest cells into CECs.

In conclusion, the high throughput expression analysis of CECs presented in this paper revealed a panel of genes (SLC4A11, COL8A2 and CYYR1) that could be used to identify CECs and proposed pathways (TGFβ and Wnt) that could be explored for expansion of CEC *in vitro* or generation of CECs *de novo*.

## Materials and Methods

### Research-grade Human Corneoscleral Tissues

Research-grade corneoscleral tissues from cadaver human donors considered unsuitable for transplantation were procured from Lions Eye Institute for Transplant and Research Inc. (Tampa, FL, USA). Overall general health of the donor before death was also considered which included previous history or medical treatment that might damage or affect the growth of the corneal endothelium. Research corneas were preserved and transported in Optisol-GS at 4°C, and were used within 13 days from preservation. The ages of donors ranged from 19–76 years (Table 11).

**Table 11:** Donor information.

Serial Number	Age	Sex	Cell Count (OS/OD)	Cause of Death					
					A	B	C	D	E
Single									
01	19	M	2625 (OS)	Overdose	•				
02	23	F	3891 (OS)	Cholecystitis	•				
03	23	F	3676 (OS)	Anoxia	•				
04	25	M	3344 (OD)	Suicide	•				
05	32	F	2625 (OS)	Liver Failure	•				
06	51	F	2865 (OS)	Sepsis		•			
07	61	M	2740 (OS)	Acute Cardiac Crisis		•			
08	57	F	2597 (OD)	Pulmonary Embolism		•			
09	59	M	2494 (OD)	Cancer		•			
10	76	F	2439 (OD)	Acute Cardiac Crisis		•			
11	19	M	2375 (OD)	Anoxia				•	
12	27	F	2762 (OS)	Multiple Blunt Trauma				•	
13	19	M	3846 (OS)	Sepsis				•	
Pairs									
14	31	M	2309 (OS)2398 (OD)	Overdose			•		
15	28	M	3096 (OS)2933 (OD)	Overdose			•		
16	47	F	2387 (OS)2451 (OD)	Cerebrovascular Accident			•		
17	19	M	2865 (OS)2717 (OD)	Pneumonia				•	
18	74	M	2114 (OS)2045 (OD)	Cardiopulmonary Arrest					•
19	34	M	2725 (OS)2801 (OD)	Sepsis			•		
20	27	F	2037 (OS)2203 (OD)	Sepsis			•		

A total of 27 donor corneas consisting of 13 single donor corneas and 7 paired donor corneas were used in this study. Donor age ranged from 19–76.

A: pooled CEC-DM from five young donors;

B: pooled CEC-DM from five old donors;

C: Isolated for cell culture;

D: young CEC-DME control;

E: old CEC-DME control.

OS: oculus sinister.

OD: oculus dexter.

### Isolation of Human Corneal Endothelial Cells

Research corneas were incubated in three washes of antibiotic/antimycotic solution in PBS, 15 minutes each. Corneoscleral rims were placed endothelial-side-up on a disposable cornea vacuum punch (Ripon, England), and mildly stabilized by the vacuum suction created. A brief 30 seconds treatment with Trypan Blue solution (0.1%) was used to delineate Schwalbe’s line. The CEC-DM layer was carefully stripped off, approximately 1 mm anterior to the Schwalbe’s line (away from the trabecular meshwork) from the posterior stroma under the dissecting microscope (Nikon, Japan).

### Culture of Human Corneal Endothelial Cells

Primary cultures of CECs were established as described in [48], and propagated with some modifications. Briefly, the CEC-DM layer was digested enzymatically in collagenase A (2 mg/ml) for at least 2 hours and up to 6 hours. This allowed full detachment of the CECs from the DM, which tended to conglomerate into tightly-packed CEC clusters. The CEC clusters were rinsed once in PBS and further dissociated in TrypLE Express (TE) for 5 minutes. Cell pellets collected after a mild centrifugation (800 g for 5 minutes) were plated in culture-ware coated with FNC coating mixture. Isolated cells were left to adhere overnight in a stabilization medium made up of Human Endothelial-SFM supplemented with 5% FBS and 1x anti-biotic/anti-mycotic. Adhered hCECs were then cultured in F99 medium containing Ham’s F12 and M199, mixed in a 1∶1 ratio, supplemented with 5% FBS, 20 µg/ml ascorbic acid, 1x Insulin-Transferrin-Selenium, 1x anti-biotic/anti-mycotic and 10 ng/ml bFGF. When the cultured cells reached 80–90% confluence, they were re-exposed to the stabilization medium for at least one week before passaging [49]. Confluent hCECs were passaged using TE, and seeded onto FNC-coated culture ware at a plating density of approximately 10,000 cells per cm^2^ for subsequent passage. All incubation and cultivation of hCECs were carried out in a humidified incubator at 37°C containing 5% CO_2_ unless otherwise stated. Fresh media were replenished every two days.

### RNA Extraction

For direct RNA extraction of donor tissues, isolated CEC-DM and corresponding corneal stroma button, punched out from the donor cornea tissue using an 8.0-mm diameter trephine, were rinsed once in PBS and placed directly into 1 ml Trizol reagent. It should be noted that although most of the corneal epithelium spontaneously sloughed off the cornea surface during the transport and process of the cornea, remnant cells of the basal corneal epithelium might still be present on the corneal stroma button used for RNA extraction. Tissues were homogenized using a hand-held homogenizer before addition of 200 ul of chloroform. After vigorous shaking, samples were spun at 13000 rpm for 15 min at 4°C. The upper phase, containing total RNA, was transferred to a new tube containing an equal volume of 70% ethanol. The resulting solution was transferred into a QIAGEN RNeasy column and procedures were performed as per manufacturer’s protocol with a DNAse digestion step incorporated.

### RNA Sequencing

#### Reverse transcription and amplification

RNA-seq libraries were prepared using an AB Demonstrated Protocol similar to the one reported in Tang *et al (2009).* For each sample, 100 pg of total RNA was reverse transcribed by SuperScript III reverse transcriptase (Cat no: 11752-250), using a poly(T) primer with a UP1 anchor sequence (50°C for 30 min followed by an enzyme inactivation step of 70°C for 10 min). The remaining free primers were removed by incubating with Exonuclease I (37°C 30 min, 80°C 25 min). Subsequently, a poly (A) tail was added to the 3′ end of the first strand cDNA by terminal deoxynucleotidyl transferase (37°C for 15 min, and 70°C for 10 min).

The resulting product was split into 4 tubes and used for the synthesis of the second strand cDNA, using a poly(T) primer with another anchor sequence (UP2) and TaKaRa Ex Taq HS, in a volume of 22 µl each (1 cycle of 95°C for 3 min, 50°C for 2 min and 72°C for 6 min). The product of each tube was then separately amplified in a volume of 41 µl, using primers UP1 and UP2 and TaKaRa Ex Taq HS (18 cycles of 95°C for 30 sec, 67°C for 1 min and 72°C for 6 min +6 sec per cycle). The amplified cDNA was combined and purified using a QIAquick PCR purification kit and eluted with 50 µl EB buffer.

The purified cDNA was re-split into 4 tubes and re-amplified with a volume of 90 µl, using 1.2 µl of template for each tube (95°C for 3 min followed by 12 cycles of 95°C for 30 sec, 67°C for 1 min and 72°C 6 min +6 sec per cycle). For this amplification NH2-UP1 and NH2-UP2 primers were used. The amplified cDNA for each sample was then combined, purified and size-selected (500 bp to 3 kb) by gel purification.

#### Library preparation

The ABI standard library preparation protocol (Chapter 2 of AB SOLiD 4 system library preparation guide - PN 4445673) was used for non-barcoded library preparation. The SOLiD fragment library barcoding kit module protocol (AB PN 4443045) was used for barcoded library preparation. The libraries were generated by using the SOLiD fragment library construction kit (PN 4443471) and the SOLiD fragment library barcoding kit (AB PN 4444836).

In brief 1 µg of amplified cDNA was sheared to approximately 165 bp, end repaired and P1, P2 adapters ligated. The product was then nick translated and amplified for 5 cycles before being size selected to be between ∼240 bp to 270 bp. The libraries were further processed using the AB SOLiD 4 system templated bead preparation guide (PN 4448378) and sequenced on 1/8th of a SOLiD slide.

#### Alignment and counting

The resulting RNA-seq reads were processed using the ABI Bioscope pipeline. The reads were aligned to the hg19 human reference genome. Gene expression was measured by counting the number of reads mapping uniquely to both strands of each gene footprint and normalized to the total uniquely mapped reads to the entire genome. The value was adjusted to show reads per million (RPM).

### Gene Expression Analysis

#### Reverse transcription

Reverse transcription was carried out using Superscript III (Cat no: 11752-250) according to manufacturer’s protocol.

#### High-throughput Quantitative Polymerase Chain Reaction (QPCR)

Fluidigm BioMark™System was used for high throughput QPCR and procedures were performed as per manufacturer’s protocol. Briefly, 5.6 ng of cDNA and 5x Taqman Gene Expression Assays (Applied Biosystems) were used per reaction and were loaded on a 48×48 Dynamic Array. Data was analyzed using BioMark™ HD System Software Suite. Expression from each gene was normalized to house-keeping genes (GAPDH, ATP6V0E1 and H2AFY) Error bars represent standard deviation from three replicates. The list of primers used can be found in Table 12.

**Table 12:** Primer list.

Gene Name	Array Probe ID	Catalog Number	Location of usage
ATP5B	Hs00969569_m1	4331182	Figure 3
CA3	Hs01013316_m1	4331182	Figure 4
COL8A2	Hs00697025_m1	4331182	Figure 3, 6
CYYR1	Hs00364793_m1	4331182	Figure 5, 6
EEPD1	Hs00286893_m1	4331182	Figure 5
ENO1	Hs00361415_m1	4331182	Figure 3
IER3	Hs00174674_m1	4331182	Figure 4
MGP	Hs00969490_m1	4331182	Figure 4
MYO	Hs00165345_m1	4331182	Figure 3
OLFML1	Hs00416948_m1	4331182	Figure 5
PLK	Hs00288354_m1	4331182	Figure 5
PTGDS	Hs00168748_m1	4331182	Figure 4
SFRP1	Hs00610060_m1	4331182	Figure 4
SLC2A1	Hs00892681_m1	4331182	Figure 3
SLC4A11	Hs00984689_g1	4351372	Figure 3, 6
TUB	Hs00163231_m1	4331182	Figure 5
ZNF16	Hs00937483_m1	4331182	Figure 5
ZNF846	Hs01394168_m1	4331182	Figure 5
GAPDH		4326317E	Housekeeping Gene
ATP6V0E1	Hs00859570_g1	4331182	Housekeeping Gene
H2AFY	Hs00191689_m1	4331182	Housekeeping Gene

List of primers used in this study.

#### QPCR

7500 Fast Real-Time PCR System (Life Technologies) was used for QPCR and procedures were performed as per manufacturer’s protocol. Briefly, 12.5 ng of cDNA and 2x Taqman Gene Expression Assays (Applied Biosystems) were used per reaction and loaded on a 96-well plate (for Life Technologies PCR system). Expression of each gene was normalized to GAPDH in the same run. Expression levels across samples were normalised to that in CEC culture (100 in CEC culture). Error bars represent standard deviation from three replicates. The list of primers used can be found in Table 12.

## Supporting Information

Table S1 RNAseqpooled_fulldataset. Full data set of RNA sequencing performed on 1) CEC-DM pooled from 5 young donors; 2) CEC-DM pooled from 5 old donors; 3) CEC cultures and 4) Corneal stroma pooled from 5 young donors.
(XLSX)

Table S2 Transporters. List of transporters (based on PANTHER classification system) and their respective RPM values for young CEC-DM, old CEC-DM, CEC cultures and corneal stroma. This table is a subset of Table S1.
(XLSX)

Table S3 Cytokine Receptors. List of cytokine receptors (based on PANTHER classification system) and their respective RPM values for young CEC-DM, old CEC-DM, CEC cultures and corneal stroma. This table is a subset of Table S1.
(XLSX)

Table S4 Transcription Factors. List of cytokine transcription factors (based on PANTHER classification system) and their respective RPM values for young CEC-DM, old CEC-DM, CEC cultures and corneal stroma. This table is a subset of Table S1.
(XLSX)

Table S5 TGF. List of components of the TGF pathway and their respective RPM values for young CEC-DM, old CEC-DM, CEC cultures and corneal stroma. This table is a subset of Table S1.
(XLSX)

Table S6 Wnt. List of components of the Wnt pathway and their respective RPM values for young CEC-DM, old CEC-DM, CEC cultures and corneal stroma. This table is a subset of Table S1.
(XLSX)
