# Supplementary figures and images for: High Throughput Gene Expression Analysis Identifies Reliable Expression Markers of Human Corneal Endothelial Cells
Source: PLoS One. 2013 Jul 2;8(7):e67546. doi: 10.1371/journal.pone.0067546 (PMC3699644; doi:10.1371/journal.pone.0067546)

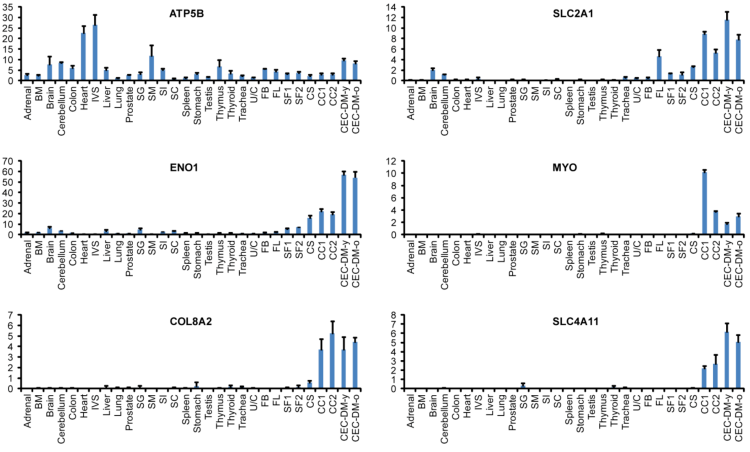

Supplement: Table S3 — Cytokine Receptors. List of cytokine receptors (based on PANTHER classification system) and their respective RPM values for young CEC-DM, old CEC-DM, CEC cultures and corneal stroma. This table is a subset of Table S1. (XLSX) [file pone.0067546.s003.xlsx]
